# Room-temperature graphitization in a solid-phase reaction

**DOI:** 10.1039/c9ra09038j

**Published:** 2020-01-03

**Authors:** Sahar Elnobi, Subash Sharma, Mona Ibrahim Araby, Balaram Paudel, Golap Kalita, Mohd Zamri Mohd Yusop, Muhammed Emre Ayhan, Masaki Tanemura

**Affiliations:** Department of Physical Science and Engineering, Graduate School of Engineering, Nagoya Institute of Technology Gokiso-cho, Showa-ku Nagoya 466-8555 Japan tanemura.masaki@nitech.ac.jp +81-52-735-5379 +81-52-735-5379; Department of Physics, Faculty of Science, South Valley University Qena 83523 Egypt sahar.elnobi@sci.svu.edu.eg; Department of Materials, Faculty of Mechanical Engineering, Universiti Teknologi Malaysia 81310 Skudai Johor Malaysia; Department of Metallurgical and Materials Engineering, Faculty of Engineering and Architecture, Necmettin Erbakan University Konya Turkey

## Abstract

Graphitized carbon including graphene has recently become one of the most investigated advanced materials for future device applications, but a prerequisite for broadening its range of applications is to lower its growth temperature. Here we report a great decrease in graphitization temperature using the well-known catalyst Ni. Amorphous carbon films with Ni nanoparticles (NPs) were deposited, using a simple one-step magnetron sputtering method, onto microgrids and a SiO_2_/Si substrate for transmission electron microscopy (TEM) and Raman spectroscopy analyses, respectively. The amorphous carbon surroundings and locations between the Ni NPs started to become graphitized during the film deposition even at room temperature (RT) and 50 °C. The graphitization was confirmed by both high-resolution TEM (HR-TEM) and Raman 2D peak analyses. The increase in the relative amount of Ni in the amorphous carbon film led to the partial oxidation of the larger Ni NPs, resulting in less graphitization even at an elevated deposition temperature. Based on the detailed HR-TEM analyses, a decreased oxidation of NPs and enhanced solubility of carbon into Ni NPs were believed to be key for achieving low-temperature graphitization.

## Abstract 2

The spontaneous graphitization for C films containing Ni NPs was attributed mainly to the increased solubility for metallic Ni NPs, and was enhanced at the deposition temperature of 50 °C.

## Graphical abstract

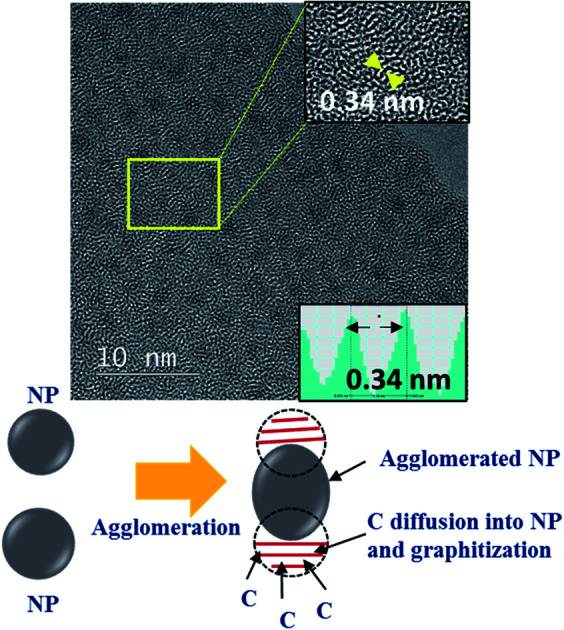

## 1. Introduction

Nowadays, the synthesis of graphene is vital for up-scaling in environment-friendly industrial applications due to its unique electronic, optical, mechanical, and thermal properties.^1,2^ But growing graphene at low temperatures is a challenge.^3–11^ For the well-known chemical vapor deposition (CVD) growth method, for instance, approaches using novel carbon sources, such as benzene, and using plasma enhancement have been proposed.^2,5,10^ For the case of the well-known catalyst Ni, the CVD temperature was reduced to 500 °C under ultra-high-vacuum conditions by tuning the growth parameters for the C_2_H_4_ carbon source.^11^

Other techniques for growing graphene besides CVD have also been pursued. Kwak *et al.* have demonstrated the formation of graphene films directly on glass and plastic substrates using graphite powders at 25–160 °C *via* the diffusion-assisted synthesis (DAS) method, where graphite powders were converted into graphene film through the diffusion of carbon along the Ni grain boundaries under quite high mechanical pressure (<1 MPa).^12^ Xiong *et al.*^13^ used magnetron sputtering to explore the mechanism of the growth of graphene from a solid carbon source through a nickel catalyst layer. They demonstrated a solid-state reaction between nickel and diffusing carbon forming a metastable nickel carbide compound at temperatures as low as 400 °C. Recently, Lu *et al.* synthesized graphene at 350 °C, by carrying out vacuum annealing of a thin film of an Ni-C-Ni “sandwich” structure on an SiO_2_/Si substrate.^6^ But growing graphene at temperatures lower than 350 °C is still challenging, especially when using Ni as the catalyst.

Very recently, Asaka *et al.* demonstrated the spontaneous local graphitization around Ni nanoparticles (NPs) even without heat treatment for amorphous carbon films deposited onto NaCl substrates pre-coated with Ni NPs.^14^ They observed the disordered graphitic structure around the Ni NPs using high-resolution transmission electron microscopy (HR-TEM). However, no trace of the Raman 2D peak was detected. Stimulated by this result, we launched a systematic investigation of the low-temperature graphitization for amorphous carbon films containing Ni NPs, which were prepared by applying a simple one-step magnetron sputtering method. The effects of temperature and Ni content on the graphitization were explored.

## 2. Experimental method

Amorphous carbon films containing Ni NPs (referred to as Ni-C films hereafter) were deposited onto commercially available microgrids (NS-M15, Okenshoji Inc.) for TEM analysis and onto SiO_2_-covered Si substrates (Ni-C/SiO_2_/Si) by using a magnetron sputtering system (SCOTT-C3 (VTR-151M/SRF), ULVAC KIKO Inc.). Porous carbon film microgrids are generally used to support fine specimens like powders for TEM. For the Ni-C film deposition, various carbon (graphite) disks, each with a diameter of 50 mm and to which a small Ni platelet had been attached (referred to as a Ni-C target hereafter), were prepared. The thickness of each Ni-C film prepared was 7 nm. In order to investigate the effect of the Ni concentration in the Ni-C film, Ni platelets of different sizes, specifically 5 × 5 and 5 × 15 mm^2^, were prepared for the attachment to the carbon disk. So, the area ratios of the Ni platelet to the carbon disk were 0.25 : 19.4 and 0.75 : 18.9, respectively. The background pressure of the chamber was 1.20 × 10^−5^ Pa and high-purity Ar (99.999%) was used as a sputtering gas at a power of 30 W for 5 minutes. In order to investigate the temperature dependence of the graphitization, some Ni-C films were deposited at RT and others at 50 °C. After the deposition of the Ni-C films was carried out, Raman spectroscopy (NRS 3300 laser Raman spectrometer) with a laser excitation energy at a wavelength of 532.08 nm and TEM (JEM ARM 200F) operated at 200 kV were used to characterize the samples.

## 3. Results

### 3.1 Films prepared from a Ni-C target with a Ni platelet area of 0.25 cm^2^

**Fig. 1:**
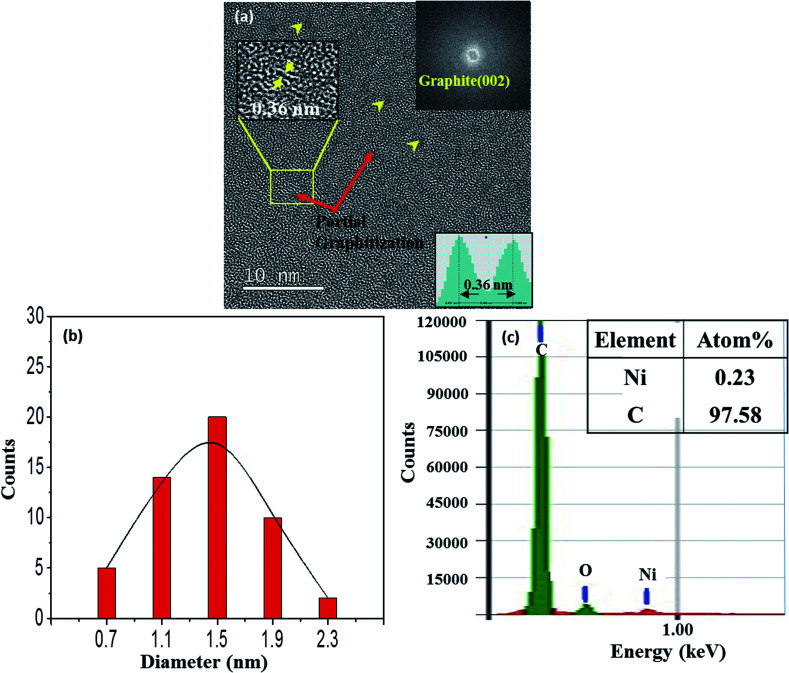
(a) TEM image of a 0.25-Ni-C film deposited on a microgrid at RT. The upper insets show an FFT image and an enlarged image of the region marked with a rectangle. The lower inset shows an intensity line profile of the part of the enlarged image (in the upper inset) marked by an arrow, and indicated an interlayer spacing of 0.36 nm. (b) Size distribution of Ni NPs in (a). (c) EDS corresponding to (a).

Some of the films were prepared by using a Ni-C target with a Ni platelet area of 0.25 cm^2^, and they are referred to as 0.25-Ni-C film and 0.25-Ni-C target, respectively. Fig. 1(a) shows a typical TEM image of a 0.25-Ni-C film deposited onto a microgrid at RT. Inspection of this image showed that the Ni-C film was featured with a dispersion of NPs with weak black contrast. Some of the NPs are indicated by arrowheads in Fig. 1(a). The upper right inset in Fig. 1(a) shows a fast Fourier transform (FFT) taken from the region of the image in Fig. 1(a) encompassed by the indicated rectangle. The FFT showed a relatively intense ring pattern attributed to graphite (002) and confirmed that the graphitization occurred at RT. Fig. 1(b) shows the size distribution of the NPs measured for Fig. 1(a), revealing that the dimensions of the NPs ranged from ∼0.7 to ∼2.3 nm with an average of about ∼1.3 nm. Since the carbon film deposited by the C disk without any Ni platelet contained no NP with black contrast, the NPs were attributed to the Ni NPs. In fact, a small amount of Ni was detected in the 0.25-Ni-C film using compositional energy dispersive X-ray spectroscopy (EDS) analysis [Fig. 1(c)]. It should be stressed that the short-range-ordered lattice fringes were discerned at many regions of the TEM image of Fig. 1(a). As shown in the lower inset in Fig. 1(a), the spacing of the ordered fringes was measured to be ∼0.36 nm, a value corresponding to the lattice spacing of graphite (002). Asaka *et al.*^14^ observed such a spontaneous local graphitization at the regions around the Ni NPs for an amorphous C film deposited on a NaCl substrate pre-coated with Ni NPs. In contrast to their observation, spontaneous local graphitization was observed also in the region between Ni NPs in our current work [see upper inset in Fig. 1(a)].

**Fig. 2:**
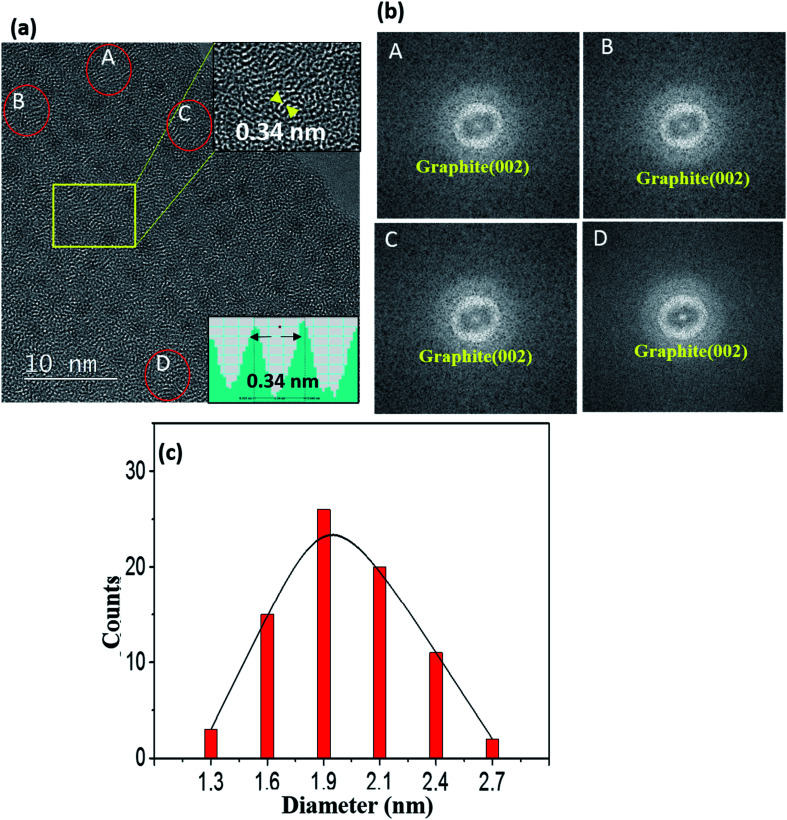
(a) TEM image of a 0.25-Ni-C film deposited at 50 °C. The upper inset in (a) shows an enlarged image of the region of the main image marked with a rectangle. The lower inset in (a) shows an intensity line profile of the part of the lattice image in the upper inset marked with an arrow, and indicated an interlayer spacing of 0.34 nm corresponding to graphite (002). (b) FFT images taken of small selected regions [A–D in (a)] of the Ni-C film. (c) Distribution of diameters of Ni NPs as measured from the TEM image shown in (a).

Fig. 2 shows a typical TEM image of a 0.25-Ni-C film deposited at 50 °C. The number density of Ni NPs of this film was less than that of the film deposited at RT. Obviously, the lattice fringes with a spacing of 0.34 nm corresponding to graphite (002), which showed short-range order, were clearly more prominent in the 0.25-Ni-C film deposited at 50 °C than in the film prepared at RT [Fig. 1(a)]. FFT images of the regions of the Ni-C film deposited at 50 °C marked with small ovals in the TEM image in Fig. 2(a) [A–D] are shown in Fig. 2(b), and each FFT image revealed a Debye ring corresponding to graphite (002). Compared with the size distribution of the particles for the film deposited at RT [Fig. 1(c)], that for the film deposited at 50 °C was slightly shifted to the larger sizes, with diameters ranging from 1.3 to 2.7 nm and an average of 2 nm, as shown in Fig. 2(c). The effects of the deposition temperature on both the graphitization and sizes of the Ni NPs are discussed below.

**Fig. 3:**
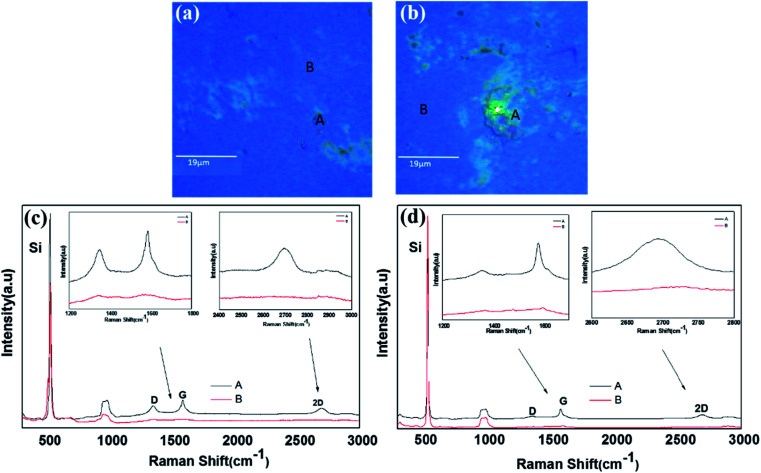
(a and b) Optical images of the 0.25-Ni-C films deposited at (a) RT and (b) 50 °C. (c and d) Raman spectra of the 0.25-Ni-C films deposited at (c) RT and (d) 50 °C, with the insets showing magnifications of the spectra in the vicinities of the D, G and 2D peaks.

In order to confirm the graphene formation (graphitization) more quantitatively, the 0.25-N-C films were also analyzed using Raman spectroscopy. Because the Raman analysis was difficult for the microgrid samples, the films simultaneously deposited onto SiO_2_-covered Si substrates were used for the Raman analyses. Fig. 3 shows their optical images and typical Raman spectra. Inspection of the optical images of the films deposited at RT and 50 °C, shown in Fig. 3(a) and (b), respectively, indicated the presence of green-colored agglomerated-like regions (referred to as region A hereafter) on blue flat surfaces (referred to as region B hereafter) for both samples. The agglomerated-like regions were more prominent for the film deposited at 50 °C. Fig. 3(c) and (d) show their Raman spectra, respectively. As seen in Fig. 3(c), region A yielded intense G and 2D peaks centered at 1583 and 2694 cm^−1^, respectively, with an intense D peak at 1349 cm^−1^, while region B yielded very weak D and G peaks at 1352 and 1581 cm^−1^, respectively, without a 2D peak. Such was also the case for the film deposited at 50 °C, as shown in Fig. 3(d). Its region A yielded G and 2D peaks centered at 1581 and 2693 cm^−1^, respectively, with a less intense D peak at 1355 cm^−1^, whereas its region B yielded small bumps in the spectra corresponding to D and G peaks at 1367 and 1595 cm^−1^, respectively, without a 2D peak. The ratio of the intensity of the D peak to that of the G peak (*I*_D_/*I*_G_) for region A of the film deposited at RT was measured to be ∼0.7 and that for the film deposited at 50 °C was measured to be 0.5. Lu *et al.* reported an *I*_D_/*I*_G_ ratio of ∼1.8 for graphene formed at 350 °C using an Ni-C-Ni thin film sandwich structure on an isolator substrate (SiO_2_/Si).^6^ The Raman spectra of the B regions of the films deposited at RT and 50 °C were quite different from that of the amorphous C film^15^ and quite similar in shape and intensity to that of the C film deposited on the NaCl substrate pre-coated with Ni NPs by Asaka *et al.*^13,16^ So, the presented work realized higher levels of graphitization at a lower temperature.

### 3.2 Films prepared from a Ni-C target with a Ni platelet with an area of 0.75 cm^2^

**Fig. 4:**
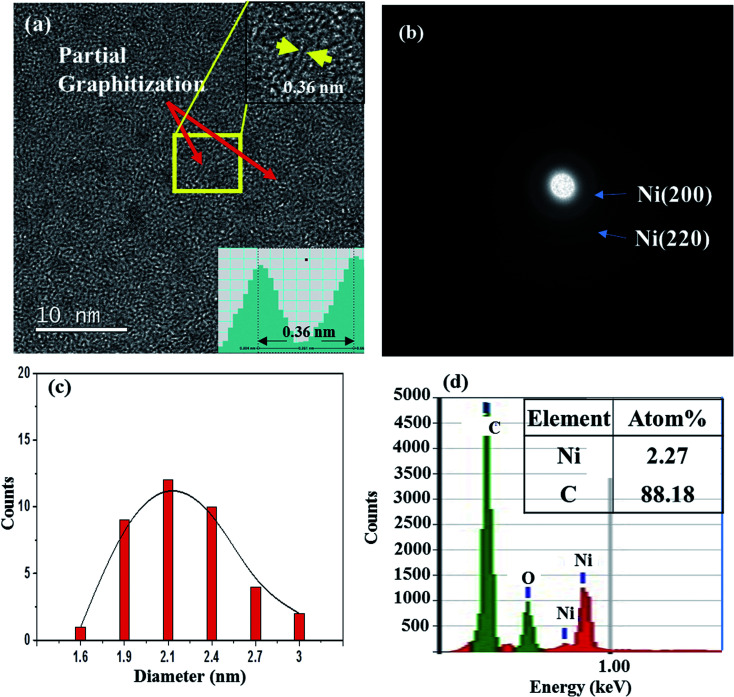
(a) TEM image of a 0.75-Ni-C film deposited on a microgrid at RT. (b) SAED pattern of this film. (c) Distribution of the diameters of Ni NPs in this film. (d) EDS spectrum of the film.

We also prepared films by using a Ni-C target with a Ni platelet area of 0.75 cm^2^, and they are referred to as 0.75-Ni-C film and 0.75-Ni-C target, respectively. These films with a higher Ni concentration than that of the 0.25-Ni-C films were prepared in order to check the effect of the Ni/C ratio. Fig. 4(a) shows a typical TEM image of a 0.75-Ni-C film deposited onto a microgrid at RT. Similar to the 0.25-Ni-C film (shown in Fig. 1), the 0.75-Ni-C film was featured by a distribution of NPs. And for this 0.75-Ni-C film, short-range graphitization with an interlayer spacing measured to be 0.36 nm was observed at many regions, with an example shown in the inset of Fig. 4(a). In contrast to the case of the 0.25-Ni-C film, NPs were dispersed more densely in the 0.75-Ni-C film and broad Debye rings corresponding to Ni (200) and Ni (220) planes were observed as shown in Fig. 4(b). Fig. 4(c) shows the distribution of diameters of Ni NPs measured from the TEM image of this 0.75-Ni-C film (Fig. 4(a)); this plot showed that the average diameter of the Ni NPs was about ∼2.1 nm, greater than that of the 0.25-Ni-C film deposited at RT. The relatively large Ni NPs would be responsible for the observed Debye rings. The greater amount of Ni was also confirmed from the acquired EDS spectrum, shown in Fig. 4(d). Also, in this spectrum, a distinct oxygen peak was also detected. This point together with the graphitization mechanism is discussed in detail below.

**Fig. 5:**
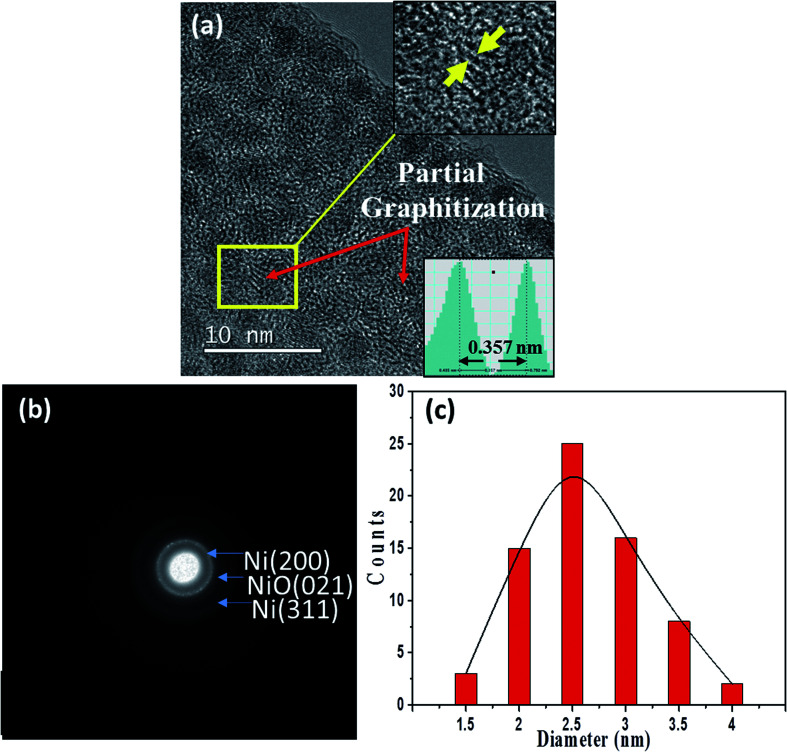
(a) TEM image of a 0.75-Ni-C film deposited at 50 °C. (b) SAED pattern of this film. (c) Distribution of the diameters of Ni NPs in this film.

Fig. 5(a) and (b) show a typical TEM image and a corresponding SAED pattern for a 0.75-Ni-C film deposited at 50 °C. Inspection of the TEM image showed an obvious dense dispersion of NPs, and lattice fringes corresponding to the graphite (002) lattice were also discerned after a careful inspection. It should be stressed that Ni oxides (weak NiO (021) Debye ring) were contained together with metallic Ni NPs in this 0.75-Ni-C film, according to its SAED pattern. Fig. 5(c) shows the distribution of diameters of the NPs measured from the TEM image of this 0.75-Ni-C film (Fig. 5(a)); this distribution revealed NPs with diameters of on average ∼2.5 nm, greater than those of the NPs of the 0.25-Ni-C films.

**Fig. 6:**
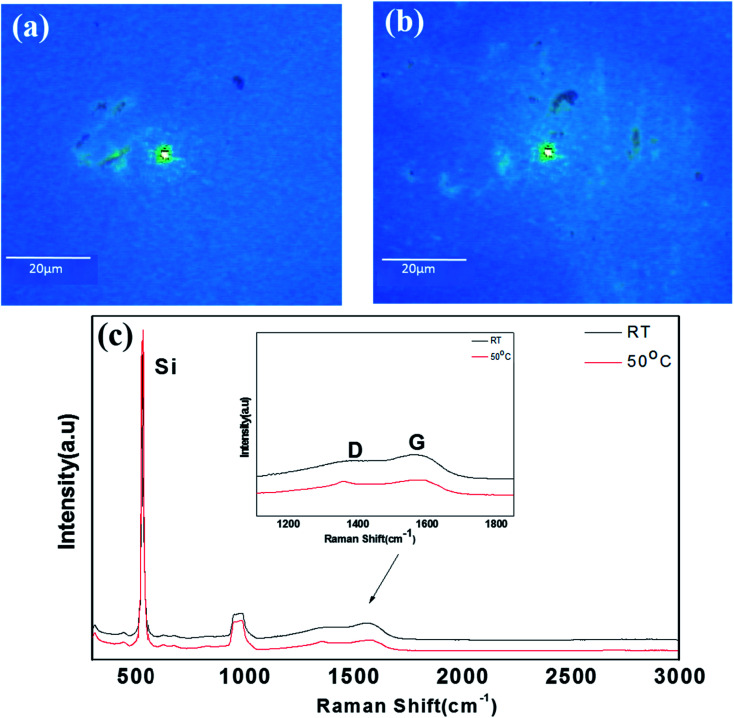
(a and b) Optical images of 0.75-Ni-C films deposited at (a) RT and (b) 50 °C. (c) Raman spectra obtained from the agglomerated-like regions of the 0.75-Ni-C films deposited at RT and 50 °C.

In order to confirm the graphitization for the 0.75-Ni-C films more quantitatively, Raman analyses were also performed on the Ni-C/SiO_2_/Si samples at RT and 50 °C. Fig. 6 shows their optical microscope images and Raman spectra. Similar to the 0.25-Ni-C films, green-colored agglomerated-like regions were observed in the optical microscope images of 0.75-Ni-C/SiO_2_/Si samples, as shown in Fig. 6(a) and (b). However, in contrast to the 0.25-Ni-C films, no distinct 2D peak was detected even from the green agglomerated-like regions for the 0.75-Ni-C films, regardless of the deposition temperature. The 0.75-Ni-C film deposited at RT yielded peaks at 1354 and 1565 cm^−1^, corresponding to D and G peaks, and the film deposited at 50 °C yielded peaks at 1356 and 1585 cm^−1^. The *I*_D_/*I*_G_ ratio of this 0.75-Ni-C film was about 0.9 regardless of the deposition temperature. These spectra were quite similar to those measured from the B regions of the 0.25-Ni-C films deposited at RT and 50 °C.

## 4. Discussion

As was found for the first time as reported above, spontaneous graphitization was enhanced at a deposition temperature of 50 °C. However, this phenomenon was prominent only for the 0.25-Ni-C film. We set out to determine the mechanism for the spontaneous graphitization, and for its enhancement at 50 °C, as well as to explain why it was prominent only for the 0.25-Ni-C film.

For the Ni-graphene system, based on the graphene growth performed under the ultra-high-vacuum conditions using single-crystal Ni, different types of graphene growth modes have been identified depending on the growth temperature.^17^ In brief, the ordered surface carbide that formed first transforms to graphene at temperatures below 500 °C, whereas the diffusion of carbon into Ni and the precipitation of graphene constitute the main mechanism of growth at high temperatures. In the latter case, the carbon solubility is an important factor.

In the present experiments, no trace of carbide was detected. So, the low-temperature growth mode of the transformation from carbide to graphene would be ruled out. The melting point of a crystal is well known to strongly depend on its size, decreasing with decreasing crystal size. So, in the present study, Ni NPs would be in the similar situation to the high temperature growth mode. In addition, the solubility of carbon in NPs generally tends to increase with decreasing NP size, because a large fraction of the C atoms would be expected to be close to the surface and the surface-to-volume ratio increases as the NP size decreases.^18–22^ Such has also been shown to be the case for Ni NPs.^21^ The high solubility of carbon into a catalyst usually readily yields multilayer graphene. Furthermore, atomic diffusion on metal surfaces for NPs is generally predicted to be much faster than for the bulk.^23,24^ These features were posited to constitute the driving force of the spontaneous graphitization observed in the current work.

**Fig. 7:**
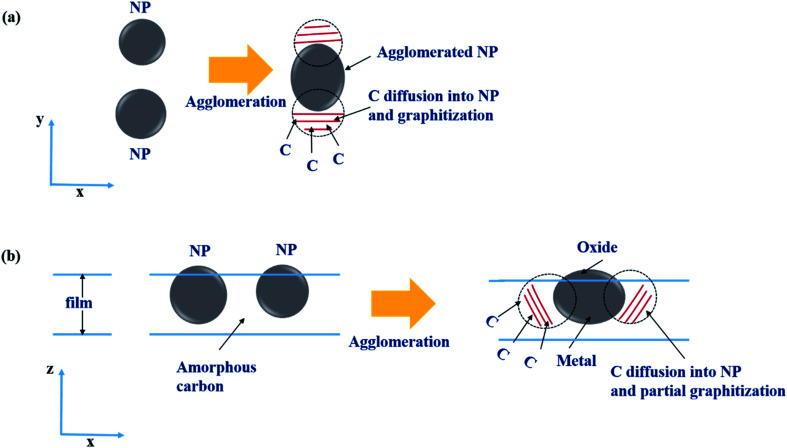
(a) Top-view and (b) side-view schematic illustrations of the graphitization in the agglomeration process during the film depositions for 0.25-Ni-C at RT and 0.75-Ni-C at 50 °C.

The enhancement of the spontaneous graphitization when the temperature was increased to 50 °C could be attributed to the solubility and diffusion rate generally increasing with increasing temperature.^25^ At the deposition temperature of 50 °C, NPs were on average slightly larger than those prepared at RT. This result may have been due to the enhanced agglomeration at the elevated deposition temperature. In this agglomeration process, the solubility of C was expected to slightly decrease with increasing NP size, yielding a graphitized layer behind the trace of moving agglomerated NPs, as depicted in Fig. 7(a). Such a mechanism may have accounted for the graphitization having occurred not only at the surroundings of the Ni NPs, but also in-between the NPs.

Such a remarkable spontaneous graphitization occurred only for the 0.25-Ni-C film. As seen in Fig. 4 and 5, larger NPs were densely dispersed in the 0.75-Ni-C films. In addition, for the 0.75-Ni-C films, an intense oxygen peak and Debye ring corresponding to Ni oxide were observed in their EDS and SAED results, respectively. Based on these observations, some parts of the NPs were thought to be out of the plane of the film and to be oxidized as depicted in Fig. 7(b). Oxidation of Ni NPs has been shown to disturb graphitization.^14^ In addition, C solubility was expected to be lower for the 0.75-Ni-C film than for the 0.25-Ni-C film, because of the larger NPs in the 0.75-Ni-C film, hence explaining the less prominent spontaneous graphitization having occurred for the 0.75-Ni-C films.

## 5. Conclusions

In conclusion, spontaneous graphitization was demonstrated from TEM and Raman spectra results for C films containing Ni NPs deposited using magnetron sputtering on SiO_2_/Si and microgrid substrates at RT and at 50 °C. The spontaneous graphitization was attributed mainly to the increased solubility for metallic Ni NPs, and was enhanced at the deposition temperature of 50 °C. For the C films containing larger and denser NPs, the accelerated spontaneous graphitization was not prominent, due to the partial oxidation of Ni NPs. Graphitization using NPs is expected to open up a new strategy for growing graphene at relatively low temperatures.

## Conflicts of interest

There are no conflicts to declare.

## Supplementary Material
